# Purification of target proteins from intracellular inclusions mediated by intein cleavable polyhydroxyalkanoate synthase fusions

**DOI:** 10.1186/s12934-017-0799-1

**Published:** 2017-11-02

**Authors:** Jinping Du, Bernd H. A. Rehm

**Affiliations:** 1 Institute of Fundamental Sciences, Massey University, Palmerston North, New Zealand; 2 Centre for Cell Factories and Biopolymers, Griffith Institute for Drug Discovery, Griffith University, Brisbane, Australia

**Keywords:** Protein purification, Polyhydroxyalkanoate, Polyhydroxyalkanoate synthase, Intein, Self-cleavage

## Abstract

**Background:**

Recombinant protein production and purification from *Escherichia coli* is often accompanied with expensive and complicated procedures, especially for therapeutic proteins. Here it was demonstrated that, by using an intein cleavable polyhydroxyalkanoate synthase fusion, recombinant proteins can be first produced and sequestered on a natural resin, the polyhydroxyalkanoate (PHA) inclusions, then separated from contaminating host proteins via simple PHA bead isolation steps, and finally purified by specific release into the soluble fraction induced by a pH reduction.

**Results:**

By translationally fusing a target protein to PHA synthase using a self-cleaving intein as linker, intracellular production of PHA beads was achieved. Upon isolation of respective PHA beads the soluble pure target protein was released by a simple pH shift to 6. The utility of this approach was exemplified by producing six target proteins, including *Aequorea victoria* green fluorescent protein (GFP), *Mycobacterium tuberculosis* vaccine candidate Rv1626, the immunoglobulin G (IgG) binding ZZ domain of protein A derived from *Staphylococcus aureus*, human tumor necrosis factor alpha (TNFα), human granulocyte colony-stimulating factor (G-CSF), and human interferon alpha 2b (IFNα2b).

**Conclusions:**

Here a new method for production and purification of a tag-less protein was developed through intein cleavable polyhydroxyalkanoate synthase fusion. Pure target protein could be easily obtained without laborious downstream processing.

**Electronic supplementary material:**

The online version of this article (10.1186/s12934-017-0799-1) contains supplementary material, which is available to authorized users.

## Background

Recombinant protein production and purification is of great significance in both academic and commercial sectors. Usually the upstream production and downstream purification are two separate processes, and the latter are often time-consuming and costly due to the requirement of expensive chromatography columns (often requiring multiple chromatography steps) or specific affinity resins which use affinity tags such as e.g. His, GST, or Strep, requiring additional protease treatment and chromatography for tag removal [1]. Similarly, in the case of medically important proteins such as human tumor necrosis factor alpha (TNFα), human granulocyte colony-stimulating factor (G-CSF) and interferon alpha-2b (IFNα2b), their production and purification from extensively used *Escherichia coli* is costly and demanding. For example, several recently published studies focus on optimizing protein refolding from inclusion bodies [2–4], and many are trying to adopt solubility-enhancing tags to avoid tedious refolding [5–8]. Hence, there is a need for a streamlined process with less complicated steps toward purification of the protein of interest.

Recently, a process for protein production and purification has been reported based on intracellularly formed polyhydroxyalkanoate (PHA) beads covalently displaying PHA synthase-target fusions [9, 10]. In these studies either an enterokinase cleavage site or a sortase plus its recognition site was inserted between PHA synthase and target protein. This enabled production of the target protein covalently bound to PHA beads. After isolation of beads the target protein could be released by adding either a protease (enterokinase) [9] or chemical reagents (CaCl_2_ and triglycine) [10].

Also recently, a pH or thiol inducible intein has been employed in combination with PHA beads for target protein production and purification [11–14]. These strategies relied on PHA phasin (PhaP) [11–13] or a PHA regulatory protein (PhaR) [14] that non-covalently associated with beads as the target protein fusion partner. The use of the pH or thiol inducible intein as linker allowed the release of target protein by a pH drop or addition of thiols. However, the non-covalently anchoring of the target protein to PHA beads caused leakage during the PHA bead wash cycles.

Here we aimed to use of the PHA synthase as fusion protein partner to covalently anchor a target protein to in vivo formed PHA beads. A self-cleavable intein served as linker between PHA synthase and target protein to enable specific release and purification of the target protein upon PHA bead isolation. To validate this new protein production and purification approach, six target proteins varying in structure and function were considered. Three of these proteins were high-value therapeutic proteins.

## Methods

### Bacterial strains and growth conditions

The *E. coli* strains and plasmids used in this study are listed in Table 1.

**Table 1:** Bacterial strains and plasmids used in this study

Strain or plasmid	Genotype or description	Source or references
*E. coli* strains		
XL1-Blue	*recA1 endA1 gyrA96 thi*-*1 hsdR17 supE44 relA1 lac* [F’ *proAB lacI* ^q^Z∆*M15* Tn*10* (Tet^r^)]	Stratagene
BL21 (DE3)	F− *ompT hsdS*_B_ (r_B_− m_B_−) *gal dcm* (DE3)	Novagen
SHuffle^®^ T7 express	*fhuA2 lacZ::T7 gene1* [lon] *ompT ahpC gal λatt:*:pNEB3-r1-*cDsbC* (Spec^R^, *lacI* ^*q*^) Δ*trxB sulA11 R(mcr*-*73::miniTn10*–Tet^S^)2 [dcm] *R(zgb*-*210::Tn10*–Tet^S^) *endA1 Δgor ∆(mcrC*-*mrr)114::IS10*	New England BioLabs
Plasmids		
pTWIN1	pBR322 derivative (Ap^r^) encoding two mini-inteins (*Ssp* DnaB and *Mxe* Gyr)	New England BioLabs
pMCS69	pBBR1MCS derivative encoding PhaA and PhaB	[15]
pMCS69E	pBBR1MCS derivative encoding Erv1p in addition to PhaA and PhaB	[22]
pET14b-ZZ(−)PhaC	pET14b derivative encoding ZZ-PHA synthase	[21]
pET14b-PhaC-linker-ZZ	pET-14b derivative encoding PhaC-linker-ZZ	[16]
pET14b-PhaC-linker-GFP	pET14b derivative encoding PHA synthase-linker-GFP	[17]
pPOLY-C-phaC-rv1626	pET14b derivative encoding PHA synthase-RV1626	[20]
pET14b-PhaC-intein-GFP	pET14b derivative encoding PHA synthase-intein-GFP	This study
pET14b-PhaC-intein-Rv1626	pET14b derivative encoding PHA synthase-intein-Rv1626	This study
pET14b-PhaC-intein-ZZ	pET14b derivative encoding PHA synthase-intein-ZZ	This study
pET14b-PhaC-intein-TNFα	pET14b derivative encoding PHA synthase-intein-TNFα	This study
pET14b-PhaC-intein-IFNα2b	pET14b derivative encoding PHA synthase-intein-IFNα2b	This study
pET14b-PhaC-intein-G-CSF	pET14b derivative encoding PHA synthase-intein-GCSF	This study

Plasmid propagation was performed in *E. coli* XL1-Blue (Stratagene, La Jolla, CA, USA) with LB broth at 37 °C, 200 rpm.

For PHA bead and protein production in flask, *E. coli* BL21(DE3) strains (Novagen, Madison, WI, USA) were first transformed with the helper plasmid pMCS69 encoding PhaA and PhaB [15] and subsequently with the fusion protein expressing plasmid. Antibiotics (100 μg/ml ampicillin and 50 μg/ml chloramphenicol) were used as appropriate.

Cultures with a starting OD600 of 0.1 (~ 2–4% inoculum depending on the growth of overnight pre-cultures) were grown at 37 °C in pH buffered Terrific Broth (12 g/l tryptone, 24 g/l yeast extract, 4 ml/l glycerol, 25 mM HEPES, pH 8.6) supplemented with 1% (w/v) glucose for about 3 h to reach an OD600 of 0.5–0.8, induced with 1 mM IPTG (isopropyl-β-d-thiogalactopyranoside), and allowed to grow at 22 °C for approximately additional 20 h. In order to counteract media acidification and the cytosolic pH drop effect during cell cultivation, an extra of 25 mM HEPES (pH 8.6) was added manually every 3 h for the first 12 h.

### Plasmid construction

Here the PHA synthase from *Ralstonia eutropha* (PhaC) is utilized as with previous PHA synthase based protein production and purification efforts [9, 10]. For plasmid pET14b-PhaC-intein-GFP, the *phaC* gene flanked by *Xba*I and *Nde*I sites was excised from plasmid pET14b-PhaC-linker-GFP [17] and inserted into the corresponding *Xba*I and *Nde*I sites on the plasmid pTWIN1 (NEB); then the resulting *phaC*-*intein* fusion gene between *Xba*I and *Xho*I sites was excised and ligated back into the corresponding *Xba*I and *Xho*I sites of the original plasmid pET14b-PhaC-linker-GFP.

Then, for plasmid pET14b-PhaC-intein-RV1626, the *rv1626* gene flanked by *Xho*I and *Bam*HI sites was excised from plasmid pPOLY-C-phaC-Rv1626 [10] and inserted into the corresponding *Xho*I and *Bam*HI sites of plasmid pET14b-PhaC-intein-GFP.

Similarly, for plasmid pET14b-PhaC-intein-ZZ, a *zz* gene from plasmid pET14b-PhaC-linker-ZZ [16] was used to replace the *gfp* gene of plasmid pET14b-PhaC-intein-GFP.

For preparation of corresponding plasmids harbouring the genes *tnfα*, *g*-*csf* or *ifnα2b*, respectively, existing sources (derivatives of sortase encoding plasmids [10]) were used to swap in the intein against the sortase gene.

Here we adopted a C-terminal fusion of PHA synthase based on the following two considerations. First, the pH inducible *Ssp* DnaB intein linker (as derived from commercial pTWIN1 vector) only cleaves at its C-terminus asparagine (Asn) site. Therefore, to achieve the release of a free target protein, the target protein has to be fused to C-terminus (Asn) of intein, which leaves the only option to start the tripartite fusions with PHA synthase, then intein linker, and then target protein. Second, to ensure bead producing functionality of PHA synthase, the hydrophobic environment at the C terminus of the synthase needs to be maintained [17]. However hydrophobicity analysis of the N-terminal regions of *Ssp* DnaB intein showed hydrophilic properties, therefore we purposely kept the CBD domain appearing before the *Ssp* DnaB intein (as derived from commercial pTWIN1 vector) to act as a hydrophobic linker between PHA synthase and *Ssp* DnaB intein.

### PHA bead isolation

Cells were harvested and re-suspended via a homogenizer (MICCRA D-9 45132, Müllheim, Germany) to a 10% slurry in lysis buffer as previously described [18], and then disrupted by using a microfluidizer (Microfluidics M-110P, Westwood, MA, USA). Beads were recovered by centrifugation at 6000×*g* for 30 min at 4 °C. Then beads were washed twice at 8000×*g* for 20 min with lysis buffer, once with a half diluted TBE buffer (20 mM Tris, 500 mM NaCl, 1 mM EDTA, pH 8.6), once with the TBE buffer, and stored as 20% slurry in TBE buffer.

### Activation of intein beads and isolation of target protein

Before bead activation, 1 ml of the 20% bead slurry was removed into a pre-weighed 1.5 ml tube, pelleted down at 8000×*g* for 4 min, washed with 1 ml of urea washing buffer (100 mM Tris, 5 mM EDTA, 1M urea, 2% v/v Triton X-100, pH 8.6) for three times to remove any residual impurities. The resulting pellet was washed once with low pH cleavage buffer (same TBE buffer but with a low pH of 6.0), weighed again, and re-suspended to a 40% slurry in cleavage buffer. The beads were incubated on a rotary mixer (Labnet Mini LabRoller, USA) at 25 °C for 16 h. To isolate the released soluble target protein, the mixture was centrifuged at 17,000×*g* for 10 min, and the supernatant was transferred into a clean tube, neutralised with high pH TBE buffer (pH 9.1) buffer, and analysed by SDS-PAGE. Protein concentration was quantified using densitometry from SDS-PAGE gels and including known standards of bovine serum albumin (BSA) (Additional file 1: Figure S1; Additional file 2: Figure S2A, B). For PHA synthase-intein-GFP beads, after supernatant collection and bead sampling, the remaining post-cleavage beads were re-suspended to a 40% slurry in cleavage buffer and subjected to a second round of cleavage. For therapeutic targets, an additional 0.2% (v/v) Tween 20 was included in the cleavage buffer to improve solubility.

### Protein identification via LC–MS/MS

In-gel digestion [19] was carried out for all cleaved target proteins before subjecting them to LC–MS/MS analysis.

### Functional assays for target proteins

For GFP protein, a FLUOstar Omega (BMG labtech, Offenburg, Germany) microplate reader was used to measure the fluorescence of eluted GFP.

For Rv1626, an anti-Rv1626 IgG [20] was diluted 1:10,000 and used for immunoblotting against total host cell proteins, all bead proteins or soluble fractions (PHA synthase-intein-GFP beads and soluble fractions were included as negative controls). Goat anti-mouse IgG HRP-conjugate (1:10,000) (Abcam ab6789, Bristol, UK) was used for detection of bound IgG antibodies.

In addition, an ELISA assay was performed in order to further investigate the antigenicity of Rv1626 as well as IgG binding function of ZZ domain. Microlon high-binding plates (Greiner Bio-One 655061, Frickenhausen, Germany) were incubated overnight at 4 °C with 50 μl of Rv1626 or ZZ at 1 ng/μl concentration in TBE buffer. And as negative and positive controls, 50 μl of wild type PHA synthase beads (with an equivalent 50 ng of protein amount in terms of PHA synthase) or ZZ-PHA synthase beads ([21], with an equivalent 50 ng of protein amount in terms of ZZ alone) respectively were included. Then primary anti-Rv1626 IgG was added only for Rv1626 and blank, while primary anti-DDA IgG [20] only for Rv1626 as a negative control, and the secondary goat anti-mouse IgG HRP-conjugate antibody to all sample wells for detection of bound IgG antibodies. A summary of ELISA reagents added, is listed in Table 2.

**Table 2:** ELISA assay to assess antigenicity of Rv1626 and IgG binding function of ZZ domain

Column	Test material	1st mouse anti-DDA IgG	1st mouse anti-Rv1626 IgG	2nd rabbit anti-mouse IgG
1	PBS blank		✓	✓
2	Rv1626 (primary anti-Rv1626 IgG)		✓	✓
3	Rv1626 (primary anti-DDA IgG)	✓		✓
4	Rv1626 (secondary antibody only)			✓
5	ZZ (secondary antibody only)			✓
6	ZZ beads (secondary antibody only)			✓
7	WT beads (secondary antibody only)			✓

For therapeutic proteins, in order to verify their antigenicity as well as their correct folding state, ELISA assays were performed with corresponding conformational antibodies applicable only for ELISA purpose. Respective protein standards and antibodies were all from Sino Biological Inc. (Beijing, PRC), namely human TNFα protein (10602-HNAE) and TNFα antibody rabbit PAb (10602-T16), human G-CSF protein (10007-HNCE) and G-CSF antibody rabbit PAb (10007-T16) as well as human interferon alpha 2 protein (13833-HNAY) and IFNα2 antibody rabbit PAb (13833-T16). Goat anti-rabbit IgG HRP-conjugate (1:3000) (Abcam ab6721) was used as secondary antibody. ELISA assay was performed as mentioned above, but overnight plate coating was carried out with 80 μl of protein sample or standard at 0.2 µg/ml concentration in TBS buffer.

## Results

### Engineering of PHA synthase-intein-target protein fusion enabled PHA bead production and facilitated purification of the target protein

**Fig. 1:**
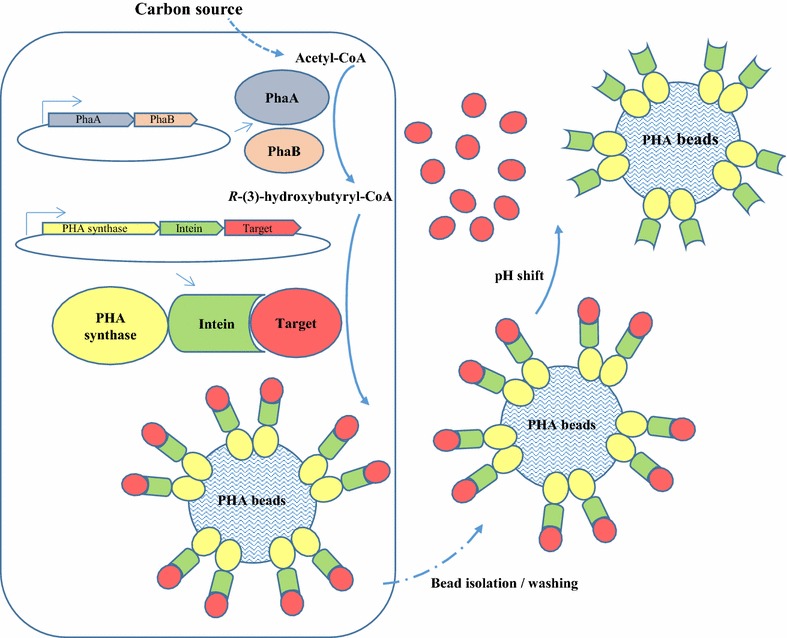
Schematic representation of recombinant protein production mediated by an intein cleavable polyhydroxyalkanoate synthase fusion as described in this study

A schematic illustration for protein purification based on intein cleavable PHA synthase fusion is shown in Fig. 1. Three proteins were first tested with this PHA synthase-intein-target protein fusion based system, including green fluorescent protein (GFP), *Mycobacterium tuberculosis* vaccine candidate antigen Rv1626, and the immunoglobulin G (IgG) binding ZZ domain of protein A derived from *Staphylococcus aureus*.

**Fig. 2:**
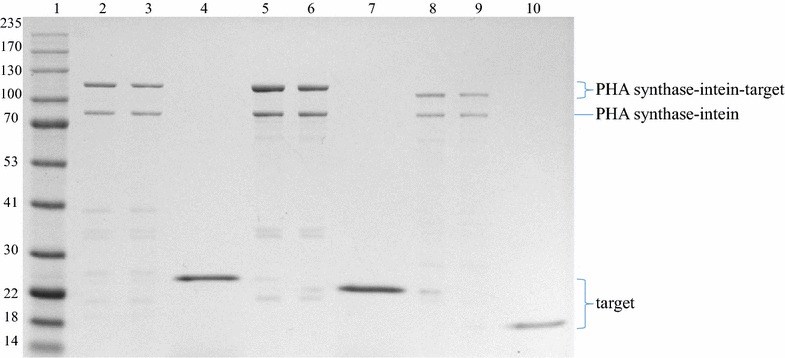
SDS-PAGE (10% Bis–Tris Gel/MOPS buffer) of the PHA beads pre- and post-cleavage, as well as the resulting soluble fractions upon a 16-h-incubation. Lane 1, molecular weight marker; lane 2, PHA synthase-intein-GFP beads pre cleavage; lane 3, PHA synthase-intein-GFP beads post cleavage; lane 4, GFP in the soluble fraction (~ 28 kDa); lane 5, PHA synthase-intein-Rv1626 beads pre cleavage; lane 6, PHA synthase-intein-Rv1626 beads post cleavage; lane 7 Rv1626 in the soluble fraction (~ 24 kDa); lane 8, PHA synthase-intein-ZZ beads pre cleavage; lane 9 PHA synthase-intein-ZZ beads post cleavage; lane 10, ZZ in the soluble fraction (~ 18 kDa)

Polyhydroxyalkanoate beads could be isolated from all strains producing the respective fusion protein, and a dominant protein band corresponding to the PHA synthase-intein-target protein was detected as associated with beads by using SDS-PAGE (Fig. 2). Moreover, in all cases, lowering the pH to 6 induced self-cleavage of intein and only the pure target protein became soluble, i.e. was released into the supernatant without any detectable contaminating proteins (Fig. 2). Gel densitometry indicated that after 16 h incubation under cleavage conditions, about 7% of PHA synthase-intein-target protein was converted into insoluble PHA synthase-intein and soluble target protein. The purified target proteins amounted to around 100 µg/g of wet beads or slightly over 10 µg/g of wet cell biomass (Table 3).

**Table 3:** Quantification and function assessment of purified model proteins

Target protein	µg per gram wet beads	µg per gram wet cell mass	Cleavage ratio (%)	Function assessment
GFP	104.9	11.6	6.6	Fluorescence as measured at excitation 395 nm/emission 509 nm
Rv1626	136.7	14.5	6.5	Antigenicity in immunoblot/ELISA assay
ZZ	95.7	14.6	7.6	IgG binding capability in ELISA assay

All of the bands corresponding to the three purified target proteins were separately excised and subjected to in-gel trypsin digestion followed by LC–MS/MS analysis, which confirmed the identity of all three purified target proteins (Additional file 3: Figure S3).

**Fig. 3:**
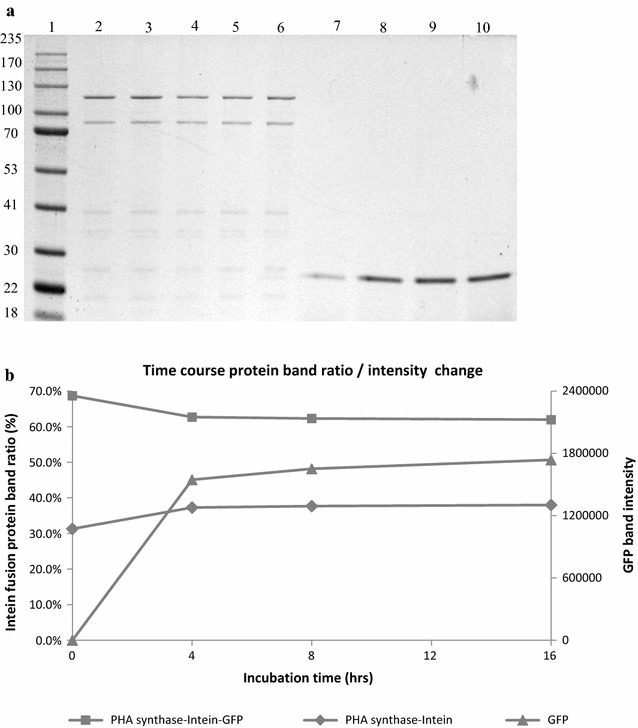
Time course GFP cleavage off PHA synthase-intein-GFP beads as shown by SDS-PAGE (**a**) and band ratio/intensity change graph of concerned proteins (**b**). Lane 1, molecular weight marker; lane 2, beads pre cleavage; lane 3, beads after 16 h incubation at pH 8.6 as control; lane 4, beads after 4 h incubation at pH 6; lane 5, beads after 8 h incubation at pH 6; lane 6, beads after 16 h incubation at pH 6; lane 7, supernatant after 16 h incubation at pH 8.6 as control; lane 8, supernatant after 4 h incubation at pH 6; lane 9, supernatant after 8 h incubation at pH 6; lane 10, supernatant after 16 h incubation at pH 6

In order to study, whether the target protein is increasingly released over continuous incubation time with the low pH buffer, we examined the cleavage of PHA synthase-intein-GFP as associated with PHA beads over a 16 h time course, and as expected, an increase in soluble GFP concentration and a shift of the PHA synthase-intein-GFP band to the PHA synthase-intein band was observed over time (Fig. 3a, b). However, prolonged incubation of 24 h did not improve the yield of GFP presumably due to degradation of the cleaved GFP (data not shown).

As significant amount of non-cleaved PHA synthase-intein-target still remained on the PHA beads even after prolonged 24 h of incubation. A further attempt was made to see whether an additional round of incubation with the low pH buffer could lead to further cleavage of the target protein. Interestingly, comparable amounts of GFP could again be cleaved off in a further round of 16 h cleavage reaction (Additional file 4: Figure S4).

### Functional assays of purified proteins

Polyhydroxyalkanoate synthase-intein-GFP displaying beads and corresponding cleavage products were green fluorescent when exposed to UV light. Fluorescence of the purified GFP was monitored and an intensity increase was observed over a 16 h cleavage cycle (Additional file 5: Figure S5). Moreover, purified GFP from the additional cleavage round showed fluorescence similar to that obtained from the first incubation reaction (data not shown). Fluorescence of GFP is indicative of functional folding throughout this production/purification process.

**Fig. 4:**
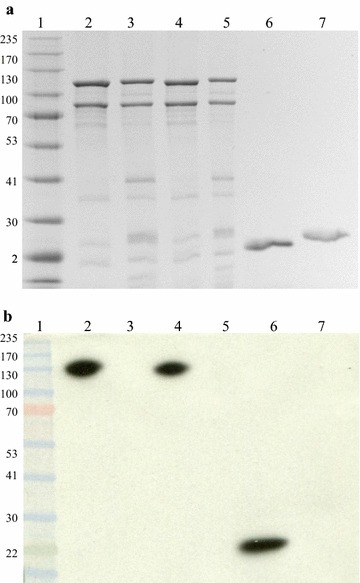
Specific recognition of the eluted Rv1626 antigen by IgG of mice immunized with beads displaying Rv1626. **a** SDS-PAGE; **b** immunoblot. Lane 1, molecular weight marker; lane 2, PHA synthase-intein-Rv1626 beads pre-cleavage; lane 3, PHA synthase-intein-GFP beads pre-cleavage as negative control; lane 4, PHA synthase-intein-Rv1626 beads post cleavage; lane 5, PHA synthase-intein-GFP beads post cleavage as negative control; lane 6, Rv1626 eluted (~ 24 kDa); lane 7, GFP eluted as negative control (~ 28 kDa)

**Fig. 5:**
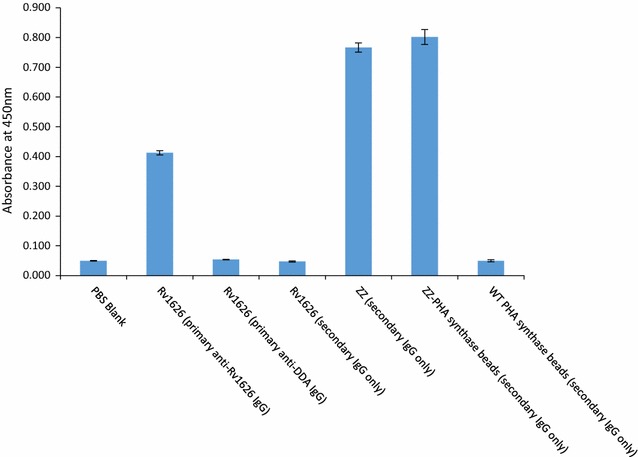
ELISA assay result showing the antigenicity of Rv1626 and IgG binding function of ZZ domain

In an immunoblot assay, an anti-Rv1626 IgG [20] specifically revealed the purified Rv1626 protein, as well as the fusion protein PHA synthase-intein-Rv1626 both in the pre-cleavage bead sample and in the post-cleavage bead sample, respectively (Fig. 4). PHA synthase-intein-GFP beads or eluted GFP did not react with the antibody and served as negative controls (Fig. 4). Further ELISA analysis proved the antigenicity of the purified Rv1626 (Fig. 5, column 2), while in the absence of the primary anti-Rv1626 antibody, only background absorbance similar to PBS was measured (Fig. 5). Both results suggested Rv1626 was produced and purified in a functional form.

The purified ZZ domain was able to bind IgG as shown in the ELISA analysis, at a similar level as compared with the positive bead control, ZZ domain displaying PHA beads, containing the same amount of ZZ protein (Fig. 5, column 5, 6), which indicated the successful retention of its IgG binding capacity when produced and purified based on the PHA synthase-intein fusion approach.

### Validation of the technique targeting real-world therapeutic proteins

To further assess the applicability of this method to produce and purify high-value therapeutic proteins, we targeted three first-line anti-cancer therapeutic proteins including human tumor necrosis factor alpha (TNFα), human granulocyte colony-stimulating factor (G-CSF), and human interferon alpha 2b (IFNα2b).

Corresponding PHA synthase-intein-target fusion-protein expressing plasmids were generated as above. PHA beads were produced and isolated as described above but using a different *E. coli* strain. Briefly, as each of these therapeutic targets contains at least one disulfide bond required for stability/functionality, a host strain providing an oxidative cytosol is necessary. Therefore, beads were produced in *E. coli* SHuffle^®^ T7 express, which has an oxidizing cytosol due to the *trxB/gor *mutations and also constitutively expresses a cytosolic form of disulfide bond isomerase (DsbC) that acts as a chaperone. Furthermore, pMCS69E was used as a helper plasmid which also encodes sulfhydryl oxidase (Erv1p) in addition to PhaA and PhaB [22].

**Fig. 6:**
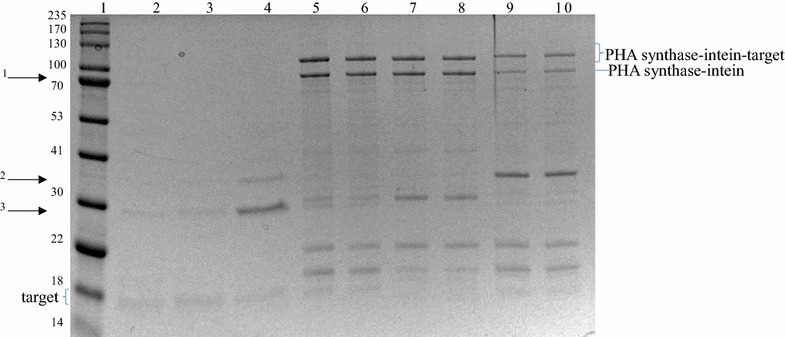
SDS-PAGE (15% Bis–Tris Gel/MOPS buffer) analysis of the resulting soluble fractions upon a 16 h incubation as well as the PHA beads pre- and post-cleavage. Lane 1, molecular weight marker; lane 2, TNFα in the soluble fraction, corresponding to the lowest bottom band (~ 18.6 kDa); lane 3, G-CSF in the soluble fraction, corresponding to the lowest bottom band (~ 19.9 kDa); lane 4, IFNα2b in the soluble fraction, corresponding to the lowest bottom band (~ 20.5 kDa); lane 5, PHA synthase-intein-TNFα beads pre cleavage; lane 6, PHA synthase-intein-TNFα beads post cleavage; lane 7; PHA synthase-intein-G-CSF beads pre cleavage; lane 8, PHA synthase-intein-G-CSF beads post cleavage; lane 9, PHA synthase-intein-IFNα2b beads pre cleavage; lane 10, PHA synthase-intein-IFNα2b beads post cleavage. Please note that in lane 2–4 there are one to three co-purified carrying-over proteins which are respectively numbered and indicated with arrows on the left side

Polyhydroxyalkanoate beads could be isolated from the respective recombinant bacteria and the PHA synthase-intein-target fusion protein could be detected as the dominant protein on the beads (Fig. 6). Levels of premature cleavage (PHA synthase-intein) were similar to that of the GFP displaying PHA beads. Activation of the beads with a pH shift to 6 released about 10–20 µg therapeutic proteins per g of wet beads or about 1–1.6 µg/g of wet cell biomass (Table 4). In all cases, therapeutic proteins were the predominant soluble proteins after cleavage; minor impurities could likely be avoided by optimization of bead isolation/washing steps or after cleavage by applying further purification steps as would be required to achieve biopharmaceutical grade purity. Furthermore, identities of all three purified therapeutic proteins were confirmed via LC–MS/MS analysis of tryptic peptides (Additional file 6: Figure S6).

**Table 4:** Quantification of purified therapeutic proteins

Target protein	µg per gram wet beads	µg per gram wet cell mass	Cleavage ratio (%)
TNFα	12.2	1.5	2.7
G-CSF	10.8	1.6	2.9
IFNα2b	18.2	1.0	1.1

**Fig. 7:**
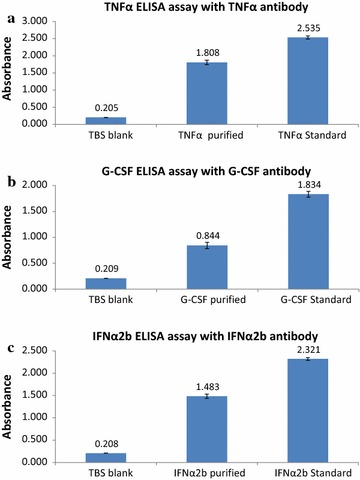
ELISA assay results with respective conformational antibody for **a** TNFα, **b** G-CSF and **c** IFNα2b showing their antigenicity as well as correct conformation

Corresponding ELISA assay results as shown in Fig. 7a–c with commercially available conformational antibodies verified their antigenicity as well as their correct folding state. A brief comparison of the results of this study versus previous methods based on the PhaP/PhaR tag is summarized in Table 5.

**Table 5:** Comparision of the PhaC based protein purification method with previous methods based on PhaP/PhaR tag

Target tag	PHA synthase-intein-target	PhaP-intein-target [11]	PhaP-intein-target [12]	PhaP-intein-target [13]	PhaR-intein-target [14]
Association with beads	Covalently attached in vivo in *E. coli*	Non-covalently attached in vivo in *E. coli*	Non-covalently attached in vivo in *R. eutropha*	In vitro non-covalent association	In vitro non-covalent association
Intein/activate condition	*Ssp* DnaB intein/low pH	∆I-CM engineered from *Mtu* recA intein/low pH	*Mxe* GyrA intein/thiols	*Ssp* DnaB intein/low pH	*Ssp* DnaB intein/low pH
Efforts on extra protein/polymer production	No concerns	Extra triple or dual PhaP protein production	Extra PhaP production	Extra separate chemical synthesis of PHB polymer	Extra separate chemical synthesis of PHB polymer
Tailoring efforts on washing/elution conditions to prevent PhaP/PhaF leaching	No concerns	Tailoring efforts needed for an intermediate salt content in washing/elution	No discussion but necessary to prevent PhaP leaching while only allow target elution	No discussion but necessary to prevent PhaP leaching while only allow target elution	No discussion but necessary to prevent PhaR leaching while only allow target elution
Limitation on purifying a potentially bead associated target	No concerns (except for PHA synthase tag itself)	With limitations, e.g. β-lactamase unsuitable	No discussion but with limitations in order to maintain non-covalent PhaP: bead binding	With limitations, e.g. PHA synthase unsuitable	No discussion but with limitations in order to maintain non-covalent PhaR:bead binding
Potentially multiple short cleavage cycles	Exemplified with PHA synthase-intein-GFP beads	Unexplored	Unexplored	Unexplored	Unexplored
Purification for therapeutic proteins containing S–S bond	Explored for TNFα, IFNα2b, and G-CSF	unexplored	Unsuitable due to use of thiols	unexplored	unexplored

## Discussion

Production of recombinant target proteins as part of a PHA synthase fusion was recently conceived as means to in vivo bind the target to PHA inclusions for facilitated purification. The target protein is via PHA synthase fusion covalently anchored to PHA beads enabling efficient enrichment of PHA beads without loss of target protein [9, 10]. In 2011, Grage et al. [9] inserted an enterokinase cleavage site between PHA synthase and the target protein, such that enterokinase treatment of respective PHA beads enabled release of the target protein. More recently Hay et al. [10] inserted a sortase and its recognition site as linker between PHA synthase and target protein, enabling the release of soluble target protein from the PHA beads upon addition of CaCl_2_ and triglycine. These platforms were advantageous for being convenient, cost-effective and folding facilitating. Nevertheless, both processes used additional reagents such as expensive proteases or fine chemicals to initiate the release of the target protein. Further downstream processing might be necessary to remove those reagents, especially when purity is of critical importance, as in the area of biopharmaceuticals or protein crystallography. This was solved here by inserting an intein between the PHA synthase and the target protein. The release of the target protein was achieved by simple pH shift of a commonly used Tris buffer, without the need for any extra protease/substrate or any associated downstream effort, which suggested it to be more suitable for a variety of medical and biochemical applications.

Previously, different PHA inclusion associated proteins (e.g. phasin, PhaP, and regulatory protein, PhaR) were utilized for protein purification by serving as PHA bead anchor fused via a self-cleavable intein to a target protein [11–14]. However, the intrinsically less stable hydrophobic interaction between the PhaP/PhaR and the PHA beads imposed certain constraints on its application. For example, salt concentration had to be tailored in order to reduce leakage of PhaP/PhaR fusions during PHA bead isolation and even during target protein elution process [11]. In addition, this approach required that target proteins do not inherently interact with PHA beads otherwise they would associate with the preformed beads rather than staying in the soluble fraction under the mild salt elution conditions [11, 13]. In contrast, in the present system, the covalently bound PHA synthase was used, which maintained the attachment of PHA synthase-intein-target to the beads regardless of salt concentrations, therefore eliminated any concerns in regard to non-elution of a potentially bead associated target or leakage under various washing/elution conditions potentially contaminating the target protein.

An additional benefit comes from the feasibility of subjecting PHA synthase-intein-target beads to multiple shorter cleavage reactions, which favoured completion of intein cleavage while minimized the risk of degradation or activity loss of the target protein, as compared to one extended cleavage reaction [14].

Apparently, the host strain background and/or the nature of the protein target affects the purity of the cleavage products, as shown by proteinaceous impurities associated with the therapeutic targets produced by the SHuffle strain but absent in model protein targets produced by the BL21 strain. This suggested the importance of aligning the strain with the target protein to minimize impurities and to enhance production of the pure target protein. Anyway, those co-purifying host cell proteins were identified via LC–MS/MS analysis as the *E. coli* chaperone protein DnaK, full length and truncated outer membrane protein A, respectively (Additional file 7: Figure S7), which are known as common impurities in *E. coli* inclusion bodies [23, 24]. Further purity and yield improvement will likely be achievable via process optimization.

It is known that protein secondary structures tend to be disrupted at acidic pH, though certain proteins remain stable under acidic conditions. For example, wild type GFP is known to be fluorescently stable over a broad pH range from 6 to 10 [25]. Also, for the solution structure of Rv1626, no change in X-ray scattering curves has been observed even when the pH dropped from 8 to 4 [26]. Moreover, a backbone amide hydrogen/deuterium exchange rate study on Z domain of staphylococcal protein A revealed that amide protons of all its three helices are protected from rapid exchange at both pH 6.5 and 4.4 demonstrating the intact structure of Z domain at acidic pH [27]. Recently researchers have developed a strategy of cation exchange chromatography at pH 6.0 for successful purification of recombinant human TNFα, and they found via circular dichroism analysis that the secondary structure of TNFα was perturbed only when pretreated below pH 5.0 [28]. Furthermore, a study on pH dependence of structural stability of G-CSF disclosed that G-CSF displayed similar secondary helical content across a pH range of 4 through 7 via CD analysis [29]. In addition, there is evidence that the highly helical secondary structure of human IFNα2b is very conserved over a broad pH range from 2 to 10 [30]. In agreement with these findings, in this study, even though all the proteins tested have been remained in an environment of pH 6 for 16 h, they are quite stable in terms of functionality/folding state. We also observed that therapeutic proteins thus purified are not 100% conformationally correct as compared to corresponding protein standard (about 73, 39 and 60% for TNFα, G-CSF and IFNα2b, respectively), this might be a reflection of the complexity of disulfide bonding with these proteins. After all, TNFα only contains one S–S bond, while both G-CSF and IFNα2b need two S–S bonds for correct folding. This is often a limiting factor when producing eukaryotic proteins in *E. coli* cells.

There are challenges remaining in working with the pH inducible *Ssp* DnaB intein. It is activated at pH 6–7 at 25 °C (New England Biolabs, NEB), while the *E. coli* cytosolic pH is documented to drop below 7 due to media acidification and acetate production during shake-flask cultivation [31]. In order to mitigate this pH drop i.e. minimize the intracellular premature cleavage of the target protein, a pH buffered Terrific broth (pH 8.6 with 25 mM HEPES) was established as PHA bead production media using a 24 h incubation time. Besides, buffers with high pH (pH 8.8) were used during cell disruption and bead isolation in order to reduce extracellular premature cleavage of the target proteins. In addition, bead production after IPTG induction was carried out at 22 °C rather than 25 °C described elsewhere [17, 20, 22, 32] to avoid activation of *Ssp* DnaB intein. This avoided undesirable premature cleavage to a level within about 30–40% as measured via densitometry analysis (Figs. 2, 6). To our knowledge, this is the first detailed effort in controlling the premature intein cleavage at shake-flask level.

Bioreactors enabling accurate real-time pH and temperature control might further reducing intracellular premature cleavage.

Furthermore, a pH buffered alkaline medium was not ideal for the growth of *E. coli* cells, which inevitably would yield less biomass, less PHA beads, and less target protein. A different host such as one of the genus *Bacillus* that could tolerant a wide range of pH could be a potential solution.

In a recently published patent application [33], a Npu split intein system showed pH sensitive cleavage without premature cleavage. Combining that Npu_*N*_ and Npu_*C*_ pair with the current PHA synthase covalent displaying platform might prove promising in producing and purifying a protein of interest.

In addition, if a target protein containing no thiol sensitive residues, unwanted premature cleavage could be eliminated through replacing the *Ssp* DnaB intein with a differently more stringently controlled one, such as a salt sensitive *Hsa* PolII intein, which cleavage is induced at > 1.5 M NaCl in the presence of reducing agent [34]. Alternatively, a thiol-inducible intein might be considered, such as *Mxe* GyrA or *Mth* RIR1 intein as provided in the commercial pTWIN1 or pTWIN 2 system (NEB).

## Conclusion

Here we demonstrated that a target protein fused to PHA synthase via a pH sensitive intein as linker mediates in vivo production of PHA beads displaying the target protein. The functional target protein could be obtained at high purity from isolated PHA beads by a pH shift to 6. Here the target protein is firstly produced as immobilized to the surface of PHA beads in vivo, then separated from contaminating host proteins via simple bead isolation steps and finally purified by specific release into the soluble fraction. This process requires neither expensive protein purification resins nor toxic chemicals or additional costly enzymes. This approach promises to serve as economic and user-friendly platform for general applications in the area of protein production and purification.

## Additional files

**Additional file 1: Figure S1.** SDS-PAGE analysis performed to assess the eluted protein content by densitometry. Protein samples were electrophoresed onto a gel, stained with Coomassie blue, an image was taken by a gel doc (BioRad Laboratories, Hercules, CA), and analysed with the IMAGE LAB software (BioRad) comparing the eluted protein with known quantities of BSA. Lane 1, Molecular weight marker; Lanes 2-6, 50, 100, 200, 400 and 500 ng BSA; Lane 7, GFP eluted (~ 28 kDa); Lane 8 Rv1626 eluted (~ 24 kDa); Lane 9 ZZ eluted (~ 18 kDa).

**Additional file 2: Figure S2.** SDS-PAGE analysis performed to assess the eluted protein content by densitometry. Protein samples were electrophoresed onto a gel, stained with Coomassie blue, an image was taken by a gel doc (BioRad Laboratories, Hercules, CA), and analysed with the IMAGE LAB software (BioRad) comparing the eluted protein with known quantities of BSA. The bottom image is an inverted version of the up one. Lane 1, Molecular weight marker; Lane 2, TNFα eluted (~ 18.6 kDa); Lane 3, G-CSF eluted (~ 19.9 kDa); Lane 4, IFNα2b eluted (~ 20.5 kDa); Lanes 5-8, 100, 200, 400 and 500 ng BSA.

**Additional file 3: Figure S3.** LC-MS/MS analysis result for the purified model proteins.

**Additional file 4: Figure S4.** SDS-PAGE analysis for PHA synthase-Intein-GFP beads / eluted GFP from two rounds of 16-h cleavage. Lane 1, Molecular weight marker; Lane 2, Beads pre cleavage; Lane 3, Beads post 1st cleavage; Lane 4, Beads post 2nd cleavage; Lane 5, GFP eluted from 1st cleavage; Lane 6, GFP eluted from 2nd cleavage.

**Additional file 5: Figure S5.** Increased active GFP cleavage over time as indicated by fluorescence measurement. Cleavage ratio is calculated as the pre- and post-cleavage difference in terms of PHA synthase Intein-GFP protein band ratio.

**Additional file 6: Figure S6.** LC-MS/MS analysis result for the purified therapeutic proteins.

**Additional file 7: Figure S7.** LC-MS/MS analysis result for the co-purified carrying-over proteins.

## Abbreviations

PHA: polyhydroxyalkanoate
GFP: green fluorescent protein
IPTG: isopropyl β-d-1-thiogalactopyranoside
HEPES: 4-(2-hydroxyethyl)-1-piperazineethanesulfonic acid)
TNFα: tumor necrosis factor alpha
G-CSF: granulocyte colony-stimulating factor
IFNα2b: interferon alpha 2b
