# Supplementary figures and images for: Purification of target proteins from intracellular inclusions mediated by intein cleavable polyhydroxyalkanoate synthase fusions
Source: Microb Cell Fact. 2017 Nov 2;16:184. doi: 10.1186/s12934-017-0799-1 (PMC5667439; doi:10.1186/s12934-017-0799-1)

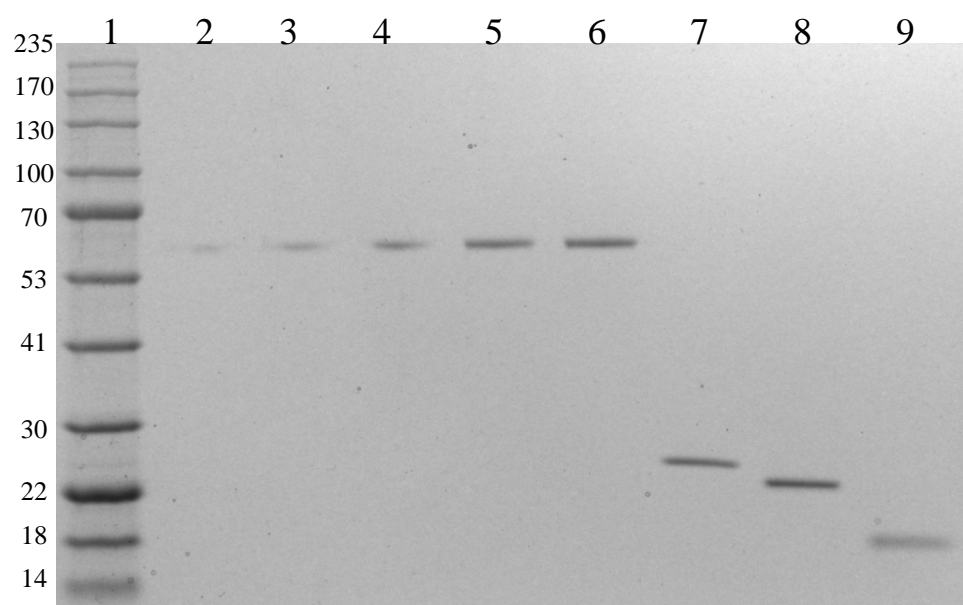

Supplement: Supplementary file 1 — Additional file 1: Figure S1. SDS-PAGE analysis performed to assess the eluted protein content by densitometry. Protein samples were electrophoresed onto a gel, stained with Coomassie blue, an image was taken by a gel doc (BioRad Laboratories, Hercules, CA), and analysed with the IMAGE LAB software (BioRad) comparing the eluted protein with known quantities of BSA. Lane 1, Molecular weight marker; Lanes 2-6, 50, 100, 200, 400 and 500 ng BSA; Lane 7, GFP eluted (~ 28 kDa); Lane 8 Rv1626 eluted (~ 24 kDa); Lane 9 ZZ eluted (~ 18 kDa). [file 12934_2017_799_MOESM1_ESM.pdf]

**A**

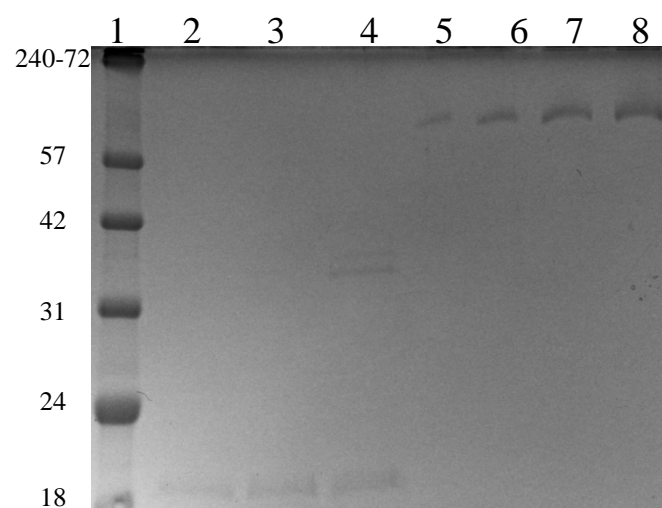

**B**

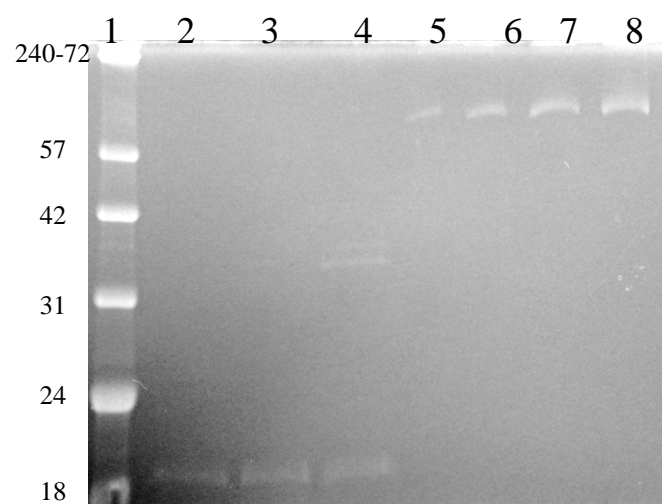

Supplement: Supplementary file 2 — Additional file 2: Figure S2. SDS-PAGE analysis performed to assess the eluted protein content by densitometry. Protein samples were electrophoresed onto a gel, stained with Coomassie blue, an image was taken by a gel doc (BioRad Laboratories, Hercules, CA), and analysed with the IMAGE LAB software (BioRad) comparing the eluted protein with known quantities of BSA. The bottom image is an inverted version of the up one. Lane 1, Molecular weight marker; Lane 2, TNFα eluted (~ 18.6 kDa); Lane 3, G-CSF eluted (~ 19.9 kDa); Lane 4, IFNα2b eluted (~ 20.5 kDa); Lanes 5-8, 100, 200, 400 and 500 ng BSA. [file 12934_2017_799_MOESM2_ESM.pdf]

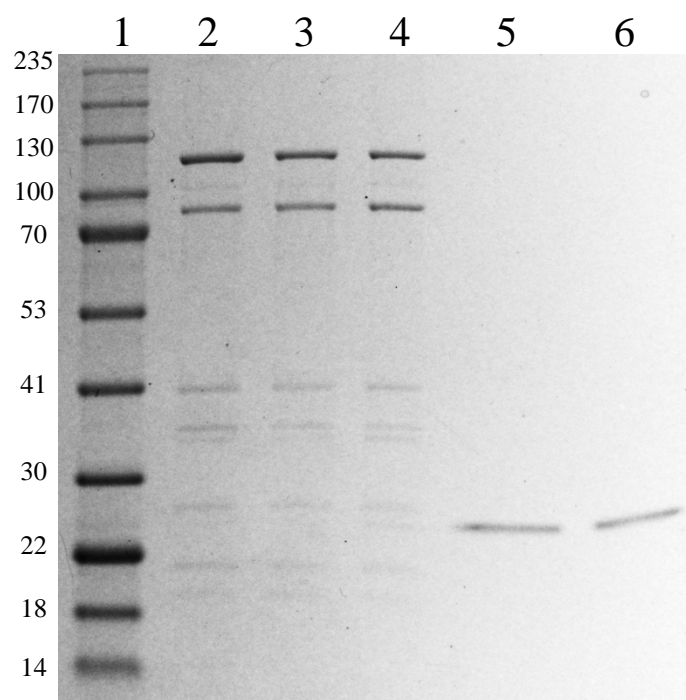

Supplement: Supplementary file 4 — Additional file 4: Figure S4. SDS-PAGE analysis for PHA synthase-Intein-GFP beads / eluted GFP from two rounds of 16-h cleavage. Lane 1, Molecular weight marker; Lane 2, Beads pre cleavage; Lane 3, Beads post 1st cleavage; Lane 4, Beads post 2nd cleavage; Lane 5, GFP eluted from 1st cleavage; Lane 6, GFP eluted from 2nd cleavage. [file 12934_2017_799_MOESM4_ESM.pdf]

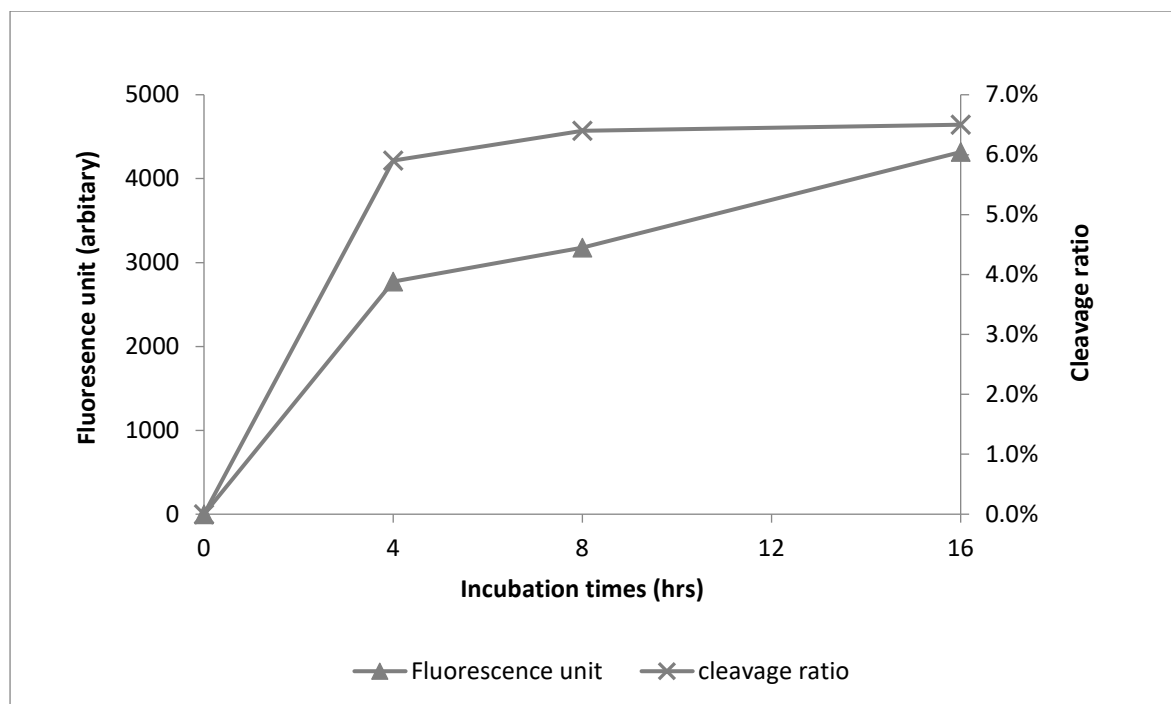

Supplement: Supplementary file 5 — Additional file 5: Figure S5. Increased active GFP cleavage over time as indicated by fluorescence measurement. Cleavage ratio is calculated as the pre- and post-cleavage difference in terms of PHA synthase Intein-GFP protein band ratio. [file 12934_2017_799_MOESM5_ESM.pdf]
