# Supplementary material for: Purification of target proteins from intracellular inclusions mediated by intein cleavable polyhydroxyalkanoate synthase fusions
Source: Microb Cell Fact. 2017 Nov 2;16:184. doi: 10.1186/s12934-017-0799-1 (PMC5667439; doi:10.1186/s12934-017-0799-1)
Supplement: Supplementary file 3 — Additional file 3: Figure S3. LC-MS/MS analysis result for the purified model proteins. [file 12934_2017_799_MOESM3_ESM.pdf]

1. *Aequorea Victoria* Green fluorescent protein

Protein sequence coverage: **70%** (166/237)

Matched peptides shown in **bold red**.

```
  1 SKGEELFTGV VPILVELDGD VNGHKFSVSG EGEGDATYGK LTLKFICTTG
 51 KLPVPWPTLV TTLTYGVQCF SRYPDHMKRH DFFKSAMPEG YVQERTIFFK
101 DDGNYKTRAE VKFEGDTLVN RIELKGIDFK EDGNILGHKL EYNYNSHNVY
151 IMADKQKNGI KVNFKIRHNI EDGSVQLADH YQQNTPIGDG PVLLPDNHYL
201 STQSALSKDP NEKRDHMVLL EFVTAAGITH GMDELYK
```

2. *Mycobacterium tuberculosis* vaccine candidate Rv1626

Protein sequence coverage: **79%** (161/205)

Matched peptides shown in **bold red**.

```
  1 MTGPTTDADA AVPRRVLIAE DEALIRMDLA EMLREEGYEI VGEAGDGQEA
 51 VELAELHKPD LVIMDVKMPR RDGIDAASEI ASKRIAPIVV LTAFSQRDLV
101 ERARDAGAMA YLVKPFSISD LIPAIELAVS RFREITALEG EVATLSERLE
151 TRKLVERAKG LLQTKHGMTE PDAFKWIQRA AMDRRTTMKR VAEVVLETLG
201 TPKDT
```

3. The immunoglobulin G (IgG) binding ZZ domain of protein A derived from *Staphylococcus aureus*

Protein sequence coverage: **59%** (68/116)

Matched peptides shown in **bold red**.

```
  1 VDNKFNKEQQ NAFYEILHLP NLNEEQRNAF IQSLKDDPSQ SANLLAEAKK
 51 LNDAQAPKVD NKFNKEQQNA FYEILHLPNL NEEQRNAFIQ SLKDDPSQSA
101 NLLAEAKKLN DAQAPK
```
