# Supplementary material for: Purification of target proteins from intracellular inclusions mediated by intein cleavable polyhydroxyalkanoate synthase fusions
Source: Microb Cell Fact. 2017 Nov 2;16:184. doi: 10.1186/s12934-017-0799-1 (PMC5667439; doi:10.1186/s12934-017-0799-1)
Supplement: Supplementary file 6 — Additional file 6: Figure S6. LC-MS/MS analysis result for the purified therapeutic proteins. [file 12934_2017_799_MOESM6_ESM.pdf]

1. Human tumor necrosis factor α (soluble form, 77-233 from original numbering)

Protein sequence coverage: **69%** (108/157)

Matched peptides shown in **bold red**.

```
  1 VRSSSRTPSD KPVAHVVANP QAEGQLQWLN RRANALLANG VELRDNQLVV
 51 PSEGLYLIYS QVLFKGQGCP STHVLLTHTI SRIAVSYQTK VNLLSAIKSP
101 CQRETPEGAE AKPWYEPIYL GGVFQLEKGD RLSAEINRPD YLDFAESGQV
151 YFGIIAL
```

2. Human Granulocyte colony-stimulating factor

(short isoform without SP or VSE after the QEKL residue, 31-65, 69-207 from original numbering)

Protein sequence coverage: **18%** (32/174)

Matched peptides shown in **bold red**.

```
  1 TPLGPASSLP QSFLLKCLEQ VRKIQGDGAA LQEKLCATYK LCHPEELVLL
 51 GHSLGIPWAP LSSCPSQALQ LAGCLSQLHS GLFLYQGLLQ ALEGISPELG
101 PTLDTLQLDV ADFATTIWQQ MEELGMAPAL QPTQGAMPAF ASAFQRRAGG
151 VLVASHLQSF LEVSYRVLRH LAQP
```

3. Human Interferon α2b (without SP, 24-188 from original numbering)

Protein sequence coverage: **40%** (66/165)

Matched peptides shown in **bold red**.

```
  1 CDLPQTHSLG SRRTLMLLAQ MRRISLFSCL KDRHDFGFPQ EEFGNQFQKA
 51 ETIPVLHEMI QQIFNLFSTK DSSAAWDETL LDKFYTELYQ QLNDLEACVI
101 QGVGVTETPL MKEDSILAVR KYFQRITLYL KEKKYSPCAW EVVRAEIMRS
151 FSLSTNLQES LRSKE
```
