# Supplementary material for: Purification of target proteins from intracellular inclusions mediated by intein cleavable polyhydroxyalkanoate synthase fusions
Source: Microb Cell Fact. 2017 Nov 2;16:184. doi: 10.1186/s12934-017-0799-1 (PMC5667439; doi:10.1186/s12934-017-0799-1)
Supplement: Supplementary file 7 — Additional file 7: Figure S7. LC-MS/MS analysis result for the co-purified carrying-over proteins. [file 12934_2017_799_MOESM7_ESM.pdf]

1. ***Escherichia coli*** **chaperone protein DnaK**

Protein sequence coverage: **67%** (427/638)

Matched peptides shown in **bold red**.

1 MGKIIGIDLG TTNSCVAIMD GTTPRVLENA EGDR**TTPSII** **AYTQDGETLV**

51 **GQPAKRQAVT** **NPQNTLFAIK** RLIGRRFQDE EVQR**DVSIMP** **FKIIAADNGD**

101 **AWVEVK**GQK**M** **APPQISAEVL** **KKMKKTAEDY** **LGEPVTEAVI** **TVPAYFNDAQ**

151 **R**QATKDAGRI AGLEVK**RIIN** **EPTAAALAYG** **LDK**GTGNRTI AVYDLGGGTF

201 DISIIEIDEV DGEK**TFEVLA** **TNGDTHLGGE** **DFDSRLINYL** **VEEFKK**DQGI

251 DLR**NDPLAMQ** **RLKEAAEKAK** **IELSSAQQTD** **VNLPYITADA** **TGPK**HMNIKV

301 TR**AKLESLVE** **DLVNR**SIEPL KVALQDAGLS **VSDIDDVILV** **GGQTR**MPMVQ

351 K**KVAEFFGK**E PR**KDVNPDEA** **VAIGAAVQGG** **VLTGDVKDVL** **LLDVTPLSLG**

401 **IETMGGVMTT** **LIAK**NTTIPT K**HSQVFSTAE** **DNQSAVTIHV** **LQGER**KRAAD

451 NK**SLGQFNLD** **GINPAPR**GMP QIEVTFDIDA DGILHVSAKD KNSGKEQKIT

501 IK**ASSGLNED** **EIQK**MVRDAE ANAEADR**KFE** **ELVQTRNQGD** **HLLHSTRKQV**

551 **EEAGDKLPAD** **DKTAIESALT** **ALETALKGED** KASIEAK**MQE** **LAQVSQKLME**

601 **IAQQQHAQQQ** **TAGADASANN** **AKDDDVVDAE** **FEEVK**DKK

2. ***Escherichia coli*** **Outer membrane protein A** (full length)

Protein sequence coverage: **82%** (283/346)

Matched peptides shown in **bold red**.

1 MKK**TAIAIAV** **ALAGFATVAQ** **AAPKDNTWYT** **GAKLGWSQYH** **DTGFINNNGP**

51 **THENQLGAGA** **FGGYQVNPYV** **GFEMGYDWLG** **R**MPYKGSVEN GAYKAQGVQL

101 TAK**LGYPITD** **DLDIYTRLGG** **MVWR**ADTKSN VYGK**NHDTGV** **SPVFAGGVEY**

151 **AITPEIATRL** **EYQWTNNIGD** **AHTIGTRPDN** **GMLSLGVSYR** **FGQGEAAPVV**

201 **APAPAPAPEV** **QTK**HFTLK**SD** **VLFNFNKATL** **KPEGQAALDQ** **LYSQLSNLDP**

251 **KDGSVVVLGY** **TDRIGSDAYN** **QGLSERRAQS** **VVDYLISK**GI PADKISAR**GM**

301 **GESNPVTGNT** **CDNVK**QR**AAL** **IDCLAPDRRV** **EIEVK**GIKDV VTQPQA

3. ***Escherichia coli*** **Outer membrane protein A**

(without SP, 22-346 from original numbering)

Protein sequence coverage: **87%** (284/325)

Matched peptides shown in **bold red**.

1 APK**DNTWYTG** **AKLGWSQYHD** **TGFINNNGPT** **HENQLGAGAF** **GGYQVNPYVG**

51 **FEMGYDWLGR** MPYK**GSVENG** **AYKAQGVQLT** **AKLGYPITDD** **LDIYTRLGGM**

101 **VWRADTK**SNV YGK**NHDTGVS** **PVFAGGVEYA** **ITPEIATRLE** **YQWTNNIGDA**

151 **HTIGTRPDNG** **MLSLGVSYRF** **GQGEAAPVVA** **PAPAPAPEVQ** **TK**HFTLK**SDV**

201 **LFNFNKATLK** **PEGQAALDQL** **YSQLSNLDPK** **DGSVVVLGYT** **DRIGSDAYNQ**

251 **GLSERRAQSV** **VDYLISK**GIP ADKISAR**GMG** **ESNPVTGNTC** **DNVK**QR**AALI**

301 **DCLAPDRRVE** **IEVK**GIKDVV TQPQA
